# Power and sample size calculations in the presence of phenotype errors for case/control genetic association studies

**DOI:** 10.1186/1471-2156-6-18

**Published:** 2005-04-08

**Authors:** Brian J Edwards, Chad Haynes, Mark A Levenstien, Stephen J Finch, Derek Gordon

**Affiliations:** 1 Laboratory of Statistical Genetics, Rockefeller University, New York, NY 10021, USA; 2 Department of Applied Math and Statistics, Stony Brook University, Stony Brook, NY 11794, USA

## Abstract

**Background:**

Phenotype error causes reduction in power to detect genetic association. We present a quantification of phenotype error, also known as diagnostic error, on power and sample size calculations for case-control genetic association studies between a marker locus and a disease phenotype. We consider the classic Pearson chi-square test for independence as our test of genetic association. To determine asymptotic power analytically, we compute the distribution's non-centrality parameter, which is a function of the case and control sample sizes, genotype frequencies, disease prevalence, and phenotype misclassification probabilities. We derive the non-centrality parameter in the presence of phenotype errors and equivalent formulas for misclassification cost (the percentage increase in minimum sample size needed to maintain constant asymptotic power at a fixed significance level for each percentage increase in a given misclassification parameter). We use a linear Taylor Series approximation for the cost of phenotype misclassification to determine lower bounds for the relative costs of misclassifying a true affected (respectively, unaffected) as a control (respectively, case). Power is verified by computer simulation.

**Results:**

Our major findings are that: (i) the median absolute difference between analytic power with our method and simulation power was 0.001 and the absolute difference was no larger than 0.011; (ii) as the disease prevalence approaches 0, the cost of misclassifying a unaffected as a case becomes infinitely large while the cost of misclassifying an affected as a control approaches 0.

**Conclusion:**

Our work enables researchers to specifically quantify power loss and minimum sample size requirements in the presence of phenotype errors, thereby allowing for more realistic study design. For most diseases of current interest, verifying that cases are correctly classified is of paramount importance.

## Background

One technique used in gene localization is the case-control genetic association study [1]. In this method, genotype and phenotype data are collected for case and control individuals [2]. Both genotype and phenotype data often contain misclassification errors [3,4], adversely affecting statistical tests used to locate disease genes [5-9]. Though phenotype misclassification has been widely studied in conjunction with disease (e.g. cancer, depression, heart disease), such studies have primarily focused on environmental association, not genetic association [10-13]. We are aware of only one recent publication considering phenotype misclassification for a test of genetic association [14].

Page et al. [3] emphasize the importance of studying phenotype errors in the context of genetic studies. They note that more than 1300 National Institutes of Health (NIH)-funded studies of complex genetic diseases have yielded fewer than 50 causative polymorphisms in humans [15,16]. More importantly, only 16%–30% of initially reported associations are confirmed without evidence of between-study heterogeneity or bias [15,17,18].

The problem of phenotype misclassification is particularly important, given the high error rates encountered in some studies. Lansbury [19] reports that postmortem pathological studies estimate that greater than 15% of Alzheimer's Disease and Parkinson's Disease cases are misdiagnosed in the clinic. Duffy et al. [12] report that in a breast cancer study conducted by Press et al. [20], nearly half (34 out of 69) of the individuals containing over expression of the immunohistochemical marker c-erbB-2 were misclassified. Burd et al. [21] found that 5%–12% of individuals previously diagnosed with Tourette syndrome were misdiagnosed. They further note that in their three-step model for linkage analysis, a 5% misclassification rate in the first step leads to a 20% error rate by the third step.

In the presence of random errors that are non-differential with respect to trait status (case or control), the type I error rate is constant [5]. That is, there is no change in significance of the classic chi-square test of independence on 2 × *n *contingency tables (the statistic of interest in this work). Here and elsewhere, *n *is the number of observed genotypes at the marker locus. However, there is a reduction in the power of the chi-square test and an increase in the minimum sample size needed to maintain constant asymptotic power at a fixed significance level [5,22,23]. A key issue that arises then is a quantification of power loss in the presence of phenotype errors.

Formulas allowing researchers to perform realistic power and sample size calculations in the presence of errors benefit researchers in the design of case-control studies by saving them the cost of excessive genotyping and phenotyping due to underpowered initial conditions. Mote and Anderson [22] computed power in the presence of what we call genotype error (although in a more general statistical setting) and proved that the power of the chi-square test of independence on *r *× *c *contingency tables (*r *= number of rows; *c *= number of columns) is always less than or equal to the power of the test when data are perfectly classified. Carroll et al. [24] developed methods for estimating the parameters of a prospective logistic model given a binary response variable and arbitrary covariates with case/control data when the covariates have measurement error. Gordon et al. [6,7] developed formulas for power and sample size calculations for the specific situation of genotype error. They used Mitra's equation for the non-centrality parameter [6,7,25] to compute the power and minimum sample size both for data with and without genotype errors. Gordon et al. [6,7] showed that a one percent increase in the sum of genotypic error rates typically results in a two to eight percent increase in the minimum sample size for the parameters and error models considered and that the increase in minimum sample size is larger when the allele frequencies are more extreme [7]. Kang et al. [8] extended this work by determining a linear approximation for the sample size increase needed to maintain constant asymptotic power at a fixed significance level. Kang et al. [8] found that (i) the cost of genotype misclassifications is a function of the true genotype frequencies and the ratio of controls to cases; (ii) in general, misclassifying a more common genotype as a less common genotype is more costly than the reverse error; and (iii) certain types of misclassification have costs that approach infinity as the minor SNP allele frequency approaches 0.

Our goal in this research is therefore two-fold: (i) to quantify power and sample size for the chi-square test of independence on 2 × *n *contingency tables in the presence of phenotype errors; and (ii) to quantify the cost of each type of phenotype error.

We present formulas to facilitate accurate power and sample size calculations in the presence of phenotype errors. We perform a genotypic test of association using the Pearson chi-square test statistic on 2 × *n *contingency tables. The degrees of freedom (in our case *n*-1) and the non-centrality parameter completely describe the power of the chi-square test. We express the non-centrality parameter in terms of the case and control sample sizes, genotype frequencies, and phenotype error model parameters. Rearranging the equation for the non-centrality parameter gives an equation for the minimum sample size. Additionally, this work extends Kang et al.'s [8] findings to the cost of phenotype errors.

## Results

As noted in the Methods section (Distinguishing case from affected and control from unaffected), we use the term *case *to refer to an individual who has been diagnosed as being affected with a given disease, whether or not that individual is truly affected. Similarly, we use the term *control *to refer to an individual who has been diagnosed as being unaffected with a given disease, whether or not that individual is truly unaffected. We use the term *affected *(respectively, *unaffected*) to refer to an individual who is truly affected (respectively, unaffected) with the disease of interest.

All notation in the Results section is defined in the Methods section (Notation).

### Design of simulation program – null and power calculations for a fixed sample size

We performed power simulations for di-allelic and tetra-allelic loci using the parameter specifications (Table 1) in the Methods section (Design of the simulation program). For the null situation, we computed the proportion of replicates for a given set of parameter specifications whose chi-square statistic exceeded the cutoff determined assuming the appropriate asymptotic null distribution (central chi-square distribution with either 2 or 9 df for di-allelic and tetra-allelic simulations, respectively). We call this proportion *the empirical significance level *for a given setting (either 5% or 1%). The median (respectively, maximum) absolute difference observed over all parameter specifications in table 1 (di-allelic and tetra-allelic) was 0.0005 (respectively, 0.002; full results not shown). That means, the empirical significance level was always within 0.002 of the significance level assuming the appropriate asymptotic null distribution. These results confirm Bross's findings [5] that non-differential phenotype misclassification does not affect the size of the chi-square test of independence.

**Table 1:** Parameter settings for null and power simulations with di-allelic and tetra-allelic loci

	Low	High
True case and control genotype frequencies	*p *= 0.05	*p *= 0.15
Pr(affected misclassified as a control) (*θ*)	0.05	0.15
Pr(unaffected misclassified as a case) (*φ*)	0.05	0.15
Disease prevalence (*K*)	0.005	0.05
Number of cases ()	500	1000
Number of controls ()	500	1000
Significance level	5%	1%
Genotype frequency parameter for tetra-allelic loci (power simulations)		
*d*	1	2

This table presents the low and high parameter settings we consider for null and power simulation calculations for di-allelic and tetra-allelic loci. As per the 2^7 ^factorial design, null and power simulations are performed on 128 distinct sets of parameter settings. Each simulation uses 100,000 iterations to determine empirical significance level (null) or simulation power. For di-allelic loci, case and control genotype frequencies are determined by the parameter *p *(see Methods – design of simulation program – power calculations for a fixed sample size). For tetra-allelic loci, genotype frequencies are determined by the parameter *d *(see Methods – Design of simulation program – power calculations for a fixed sample size).

For the power simulations, we compared the asymptotic power with the simulation power using absolute difference. That is, the absolute difference in power, defined as |simulation power - asymptotic power|, was calculated for each simulation. In table 2, we report the minimum, 10^th ^percentile, 25^th ^percentile, median, 75^th ^percentile, 90^th ^percentile, and maximum differences at the 5% and 1% significance levels. There were 2^7 ^= 128 data points for each simulation. For the majority of simulations, the absolute difference was very small. For both di-allelic loci and tetra-allelic loci at both significance levels, the median absolute difference was 0.001. For di-allelic loci, the maximum absolute difference observed was 0.012 (at the 1% significance level) while for the tetra-allelic loci, the maximum absolute difference was 0.011 (also at the 1% significance level).

**Table 2:** Percentiles for absolute difference between asymptotic power and simulation power

	5% significance level	1% significance level
	Di-allelic locus	
Minimum	0.0000	0.0000
10^th ^percentile	0.0002	0.0002
25^th ^percentile	0.0005	0.0004
50^th ^percentile	0.0010	0.0011
75^th ^percentile	0.0028	0.0026
90^th ^percentile	0.0065	0.0057
Maximum	0.0099	0.0119
	Tetra-allelic locus	
Minimum	0.0000	0.0000
10^th ^percentile	0.0000	0.0000
25^th ^percentile	0.0007	0.0008
50^th ^percentile	0.0012	0.0014
75^th ^percentile	0.0028	0.0032
90^th ^percentile	0.0072	0.0081
Maximum	0.0102	0.0111

Power simulations are performed at 100,000 iterations for each set of parameter specifications in the Methods section. Here we report various percentiles of the absolute difference |simulation power - asymptotic power| for our simulations. For each locus type (di-allelic, tetra-allelic), percentiles are computed using 2^7 ^= 128 settings documented in table 1.

Although the asymptotic power is a good enough approximation to the simulation power so that it can be used for design purposes, this difference is somewhat larger than would be expected in the event that the simulated power followed a binomial variation with probability equal to the asymptotic power (based on computation of 95% confidence intervals – results not shown). We discuss this issue below (see Discussion).

### Cost functions

Using the mathematics presented in the Methods section (Cost functions), we compute the following formulas:



In table 3, we present the values of these cost coefficients for the parameters considered in table 1. One finding becomes immediately clear. It is that the cost of misclassifying an unaffected as a case is much larger than the cost of misclassifying an affected as a control. For example, for a disease prevalence *K *= 0.05, the minimum cost coefficient *C*_*φ *_regarding misclassification of an unaffected as a case is approximately 40, occurring when R* = 2 and *p *= 0.15. The maximum cost coefficient *C*_*θ *_for the same prevalence is 0.10, occurring for the same values of R* and *p*.

**Table 3:** Cost coefficients for different types of misclassification

*K*	R*	*p*	*C*_*θ*_	*C*_*φ*_
0.005	0.5	0.05	0.01	540.29
		0.15	0.01	458.99
	1	0.05	0.01	478.32
		0.15	0.01	432.67
	2	0.05	0.01	440.18
		0.15	0.01	415.60
0.05	0.5	0.05	0.09	51.59
		0.15	0.10	43.82
	1	0.05	0.08	45.67
		0.15	0.10	41.31
	2	0.05	0.08	42.03
		0.15	0.10	39.68

The column heading for this table are as follows: *K *= prevalence; R* = ratio of controls to cases; *p *= SNP minor allele frequency in affected population; *C*_*θ *_= Cost coefficient corresponding to misclassification parameter *θ *– this is a lower bound of the percent increase in sample size necessary to maintain constant asymptotic power for every 1% increase in *θ C*_*φ *_= Cost coefficient corresponding to misclassification parameter *φ *– this is a lower bound of the percent increase in sample size necessary to maintain constant asymptotic power for every 1% increase in *φ*. The cost coefficients are computed using equation (1).

When the prevalence *K *= 0.005, the cost coefficient *C*_*φ *_becomes larger by an order of magnitude. The minimum value of *C*_*φ *_is 415, occurring as above when R* = 2 and *p *= 0.15. That means that a 1% increase in the value of *φ *requires *at least *a 415% increase in cases and controls to maintain the same power at any significance level.

A second finding that becomes clear from studying equation (1) is that the cost coefficient *C*_*φ *_has an infinite limit as the prevalence *K *approaches 0 (for any set of fixed values of the other parameters), while the cost coefficient *C*_*θ *_has a limit of 0. This results comes from the observation that the dominating terms for the cost coefficients *C*_*φ *_and *C*_*θ *_in equation (1) are (1 - *K*)/*K *and *K*/(1 - *K*), respectively.

It should be noted that the linear Taylor approximation is not very accurate for even small values of *φ*. The linear Taylor approximation is useful, though, in that it serves as a *lower *bound for the percentage sample size increase. That is, percent increase in sample size is *at least C*_*φ *_for any value of *φ*. We illustrate this point in the next section.

### Minimum sample size requirements in presence of phenotype misclassification – Alzheimer's disease ApoE example

**Figure 1:**
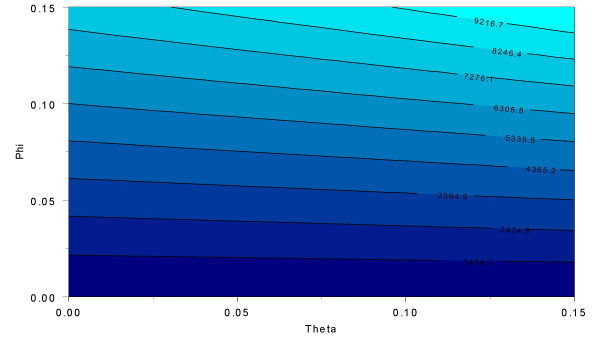
**Contour plot of minimum number of cases needed to maintain constant asymptotic power of 95% at a 5% significance level in the presence of phenotype misclassification for Alzheimer's disease ApoE example**. We compute the increase in minimum cases () needed to maintain constant 95% asymptotic power at the 5% significance level (using a central *χ*^2 ^distribution with 5 degrees of freedom) in the presence of errors. Sample sizes are computed using equation (3). The affected and unaffected genotype frequencies are taken from a previous publication [9, 14]. In that work, the marker locus considered was ApoE and the disease phenotype was Alzheimer's disease. We use the LRT_ae _estimates from table 5 of that work [9]. Six genotypes are observed in most populations. The frequencies we use to perform the sample size calculations in figure 1 are presented in the Methods section (Minimum sample size requirements in presence of phenotype misclassification – Alzheimer's Disease ApoE example). We assume that equal numbers of cases and controls are collected. Also, we specify a prevalence K = 0.02, which is consistent with recent published reports for Alzheimer's Disease in the U. S. [32]. Sample sizes are calculated for each misclassification parameter *θ*, *φ *ranging from 0.0 to 0.15 in increments of 0.01. The number of cases ranges from 484 when *θ *= *φ *= 0 to 10,187 when *θ *= *φ *= 0.15. In this figure, each (approximately) horizontal line represents a constant sample size as a function of the misclassification parameters *θ *and *φ*. For two consecutive horizontal lines, the values in between those lines (represented by different colors) have sample sizes that are between the sample sizes indicated by the two horizontal lines.

Figure 1 presents a contour plot of the minimum sample size necessary to maintain a constant power of 95% at the 5% significance level using the parameter values taken from the methods section (see Methods – Minimum sample size requirements in presence of phenotype misclassification – Alzheimer's disease ApoE example). Each approximately horizontal line represents a constant minimum number of cases (as a function of the misclassification parameters *φ *and *θ*). For two consecutive horizontal lines, the values in between those lines (represented by different colors) have sample sizes that are between the sample sizes indicated by the two horizontal lines. For example, consider the consecutive, approximately horizontal lines labeled 3394.9 and 4365.9 (third and fourth lines up, respectively, in figure 1). All values of *θ *and *φ *whose Cartesian coordinate(*θ*, *φ*) lies between these two lines have a corresponding minimum sample size  between 3395 and 4365. An example of such a pair is the coordinate (0.00,0.075). Note that the minimum sample size  of 484 occurs when *φ *= *θ *= 0 and the maximum sample size  of 10,187 occurs when *φ *= *θ *= 0.15.

Our results for the cost functions are consistent with the findings here. For values of *φ *less than 0.02, sample size increase appears to be constant in the parameter*θ*. That is, misclassification of an affected as a control does not affect the sample size estimates at all. However, even a 1% misclassification of an unaffected as a case requires a sample size increase from 486 to 921 (*φ *= 0.01, *θ *= 0.0 in figure 1; exact results not shown) to maintain constant power, an approximately 90% increase. As the probability of misclassifying an unaffected as a case *φ *increases, there appears to be an interaction between the two misclassification parameters, requiring even larger sample size increases than would be expected if the sample size increase were linear in each misclassification parameter (figure 1).

### Comparison of power loss for fixed sample size when only one misclassification parameter is non-zero

**Figure 2:**
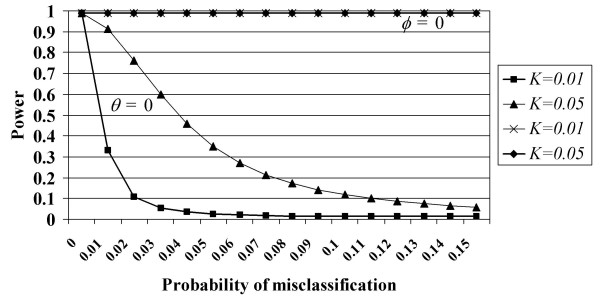
**Power to detect association for two different settings of prevalence when only one phenotype misclassification parameter is non-zero**. In this figure, the horizontal axis refers to the misclassification probability for one parameter when the second parameter is 0. For example, the graphs labeled "*φ *= 0" provide power calculations at two settings of disease prevalence (*K *= 0.05, *K *= 0.01) as a function of *θ *values ranging from 0.0 to 0.15 on the horizontal axis. Similarly, the graphs labeled "*θ *= 0" provide power calculations at two settings of disease prevalence (*K *= 0.05, *K *= 0.01) as a function of *φ *ranging from 0.0 to 0.15 on the horizontal axis.

Another way of interpreting cost is by considering the power loss for fixed sample size. We demonstrate this point in figure 2. In that figure, we present the power in the presence of phenotype misclassification when either the *θ *or *φ *parameter is set to 0 and the other parameter ranges from 0 to 0.15 in increments of 0.01. Power is calculated at the 1% significance level assuming 250 cases and 250 controls, a SNP locus with case minor allele frequency 0.05, control minor allele frequency 0.15 (Hardy Weinberg equilibrium in both populations), and two settings of disease prevalence (*K *= 0.05, 0.01). Power is determined through calculation of the non-centrality parameter (equation (2)).

The results of figure 2 further illustrate the importance of distinguishing between the two types of misclassification. When the *φ *parameter is 0, the asymptotic power is virtually independent of the value of the *φ *parameter and the disease prevalence *K*. Power values for all settings of *φ *and *K *are approximately 99%. When the *θ *parameter is 0, the asymptotic power reduces to 91% when *φ *= 0.01, *K *= 0.05 and to 33% when *φ *= 0.01, *K *= 0.01. When *φ *= 0.02, power reduces to 76% when *K *= 0.05 and to 11% when *K *= 0.01. These examples further document the dominating effect that disease prevalence has on power and/or sample size requirements in the presence of phenotype misclassification error.

## Discussion

As we noted above (Results – Design of simulation program – power calculations for a fixed sample size), the asymptotic power is a good enough approximation to the simulation power so that it can be used for design purposes. However, the difference is somewhat larger than would be expected in the event that the simulated power followed a binomial variation with probability equal to the asymptotic power. One possible explanation may be that our simulation studies were "under-powered" so that the asymptotic theory did not hold. Indeed, the median power value at the 5% significance level for our simulation studies (table 1) was 13% (full results not shown). Given such low overall power levels and also the fact that, for the SNP minor allele frequency of 0.05, Cochran's condition of a minimal expected cell count of 5 is not achieved [26], it is conceivable that effective sample sizes are not sufficient for power values based on asymptotic theory to hold. Other authors studying misclassification error have also observed this phenomenon [27].

While we have considered a genetic model-free framework here, we note that our work easily extends to a genetic model-based framework as well [6,7]. We will implement calculations using a genetic model-based framework in our web tool (next paragraph).

Given the accuracy of our method (absolute errors no larger than 0.012, based on simulations), we conclude that researchers may use our method to accurately determine power and sample size calculations for case/control genetic association studies in the presence of phenotype misclassification. We have developed a web tool that performs these calculations online. The URL for this tool is: .

## Conclusion

In this work, we developed a method for performing realistic power and sample size calculations in the presence of phenotype errors. Simulation results suggest that our formulas (equations (2) and (3)) may be used to design case/control genetic association studies incorporating phenotype misclassification. We confirmed that phenotype misclassification always reduces the power of the chi-square test of association (as was first shown by Bross [5]), and consequently, increases the minimum sample size needed to maintain constant asymptotic power.

Our cost calculations reveal two significant findings. The first is that power and/or sample size is most significantly altered by a change in disease prevalence. Specifically, the cost coefficient for misclassifying an affected as a control is of the order of magnitude *K*/(1 - *K*) and the cost coefficient for misclassifying an unaffected as a case is of the order of magnitude (1 - *K*)/*K*, where *K *is the disease prevalence (equation (1)). This finding suggests that, for many diseases of current interest, where prevalence is usually less than or equal to 0.10, *it is much more important to insure that cases are truly cases rather than controls being truly controls*. Zheng and Tian [14] made this same observation (without the explicit computation of cost coefficients) for the linear test of trend applied to cases and controls genotyped at a SNP marker.

## Methods

### Distinguishing case from affected and control from unaffected

Throughout this work, we use the term *case *to refer to an individual who has been diagnosed as being affected with a given disease, whether or not that individual is truly affected. Similarly, we use the term *control *to refer to an individual who has been diagnosed as being unaffected with a given disease, whether or not that individual is truly unaffected. We use the term *affected *(respectively, *unaffected*) to refer to an individual who is truly affected (respectively, unaffected) with the disease of interest. A key assumption we make through the paper is that we collect only cases and controls for our test of genetic association.

### Notation

We use the following notation:

#### Count parameters

*a *= Number of alleles at the marker locus. The number of genotypes at the marker locus is always *a*(*a *+ 1)/2 = *n*.

 = Number of cases; this quantity is a fixed parameter in our design.

 = Number of controls; this quantity is a fixed parameter in our design.

 = Ratio of controls to cases.

#### Probability parameters

*K *= Prevalence of disease.

*p*_0*j *_= Frequency of genotype *j *at the marker locus for the affected group, 1 ≤ *j *≤ *a*(*a*+1)/2.

*p*_1*j *_= Frequency of genotype *j *at the marker locus for the unaffected group, 1 ≤ *j *≤ *a*(*a*+1)/2.

 = Frequency of genotype *j *at the marker locus for the case group, 1 ≤ *j *≤ *a*(*a*+1)/2.

 = Frequency of genotype *j *at the marker locus for the control group, 1 ≤ *j *≤ *a*(*a*+1)/2.

#### Error model parameters

*θ *= Pr (affected individual classified as control) = 1 - *Se*, where *Se *is the sensitivity of the phenotype measurement instrument.

*φ *= Pr (unaffected individual classified as case) = 1 - *Sp*, where *Sp *is the specificity of the phenotype measurement instrument. This notation was used by Bross [5].

A key assumption we make here is that these errors are random and independent. Furthermore, they are non-differential with respect to a particular genotype [14].

#### Cost parameters

*C*_*θ *_= Cost of misclassifying an affected individual as a control. This value is the percent increase in minimum sample size necessary to maintain constant power for every one percent increase in the value of *θ*.

*C*_*φ *_= Cost of misclassifying an unaffected individual as a case. This value is the percent increase in minimum sample size necessary to maintain constant power for every one percent increase in the value of *φ*.

### Expressing case and control genotype frequencies in terms of affected and unaffected genotype frequencies

We comment that the case and control genotype frequencies, ,, may be written in terms of the affected and unaffected genotype frequencies, *p*_0*j*_, *p*_1*j*_, the disease prevalence *K*, and the misclassification error probabilities, *θ *and *φ*. Using the law of total of probability, we have:

 = [*p*_0*j *_(1 - *θ*) *K *+ *p*_1*j*_*φ*(1 - *K*)]/[(1 - *θ*) *K *+ *φ*(1 - *K*)], 1 ≤ *j *≤ *a*(*a *+ 1)/2

 = [*p*_0*j*_*θK *+ *p*_1*j*_(1 - *φ*)(1 - *K*)]/[*θK *+ (1 - *φ*)(1 - *K*)]. 1 ≤ *j *≤ *a*(*a *+ 1)/2

For a derivation, see the Appendix.

It is interesting to note that determination of case and control genotype frequencies in the presence of only phenotype error differs from determination of the same frequencies in the presence of only genotype error in that one needs to specify disease prevalence for phenotype error (in addition to specifying the respective misclassification probabilities for phenotype and genotype) [7,14].

### Test statistic for genotypic association

The test statistic considered in this work is Pearson's chi-square statistic on 2 × *n *contingency tables. Here, the two rows refer to the two possible classifications (case or control) and the *n *columns correspond to the *n *different genotypes, where *n *= *a*(*a *+ 1)/2. Using this statistic on 2 × *n *contingency tables, we test for association between genotype and disease status. We selected the genotypic test of association because the null distribution of the allelic test of association cannot be determined when either the case or control group genotype frequencies deviate from Hardy-Weinberg Equilibrium (HWE) [28,29]. Let *G*_*rc *_equal the observed count of the *c*^th ^genotype in the *r*^th ^group, where 1 ≤ *c *≤ *n *and *r *= 0 for the case population and *r *= 1 for the control population. Then, the chi-square statistic is given by the formula .

In this expression, the expected cell count of the *c*^th ^genotype in the *r*^th ^group, *E*_*rc*_, is determined by the equation *E*_*rc *_= *S*_*r*_*D*_*c*_/*N*, where  is the row total for the *r*^th ^group,  is the column total for the *c*^th ^genotype, and  is the total sample size.

Under the null hypothesis of no association between the marker locus and the disease (*p*_0*j *_= *p*_1*j *_for all *j*), the statistic *X*^2 ^is asymptotically distributed as a central *χ*^2 ^with *n *- 1 degrees of freedom. We verify this statement in our simulations (see Results).

### Asymptotic power calculations

In this section, we describe our method for computing asymptotic power in the presence of errors. The asymptotic power is summarized by a non-centrality parameter *λ*, which is a function of the case and control sample sizes and the respective genotype frequencies.

The asymptotic power is , where *β *is the probability of a type II error (accepting a false null hypothesis) and  is the cumulative distribution function (CDF) for the non-central *χ*^2 ^distribution with *n*-1 degrees of freedom evaluated at the *α *percentile of the null distribution, which is a central *χ*^2 ^distribution with *n *- 1 degrees of freedom.

### Asymptotic non-centrality parameter

Mitra [25] derived the asymptotic power function for the chi-square test for unmatched cases and controls. Under the alternative hypothesis, the distribution is a non-central *χ*^2 ^with *n *-1 degrees of freedom and non-centrality parameter λ*. Mitra [25] showed that for perfectly classified data (i.e., *θ *= *φ *= 0)), the non-centrality parameter is given by



where the sample sizes  and  are fixed by design and the genotype frequencies  and  are equal to *p*_0*j *_and *p*_1*j *_respectively, for each *j*. In the presence of phenotype errors, the genotype frequencies  and  are biased away from their true values, as indicated by formula (1). We verify the accuracy of the non-centrality parameter formula (2) using simulations (see Methods – Design of simulation program – null and power calculations for a fixed sample size).

### Increase in minimum sample size

We determine the minimum sample size needed to maintain constant power at a fixed significance level in the presence of phenotype errors. The minimum sample size for cases  can be found by rearranging equation (2) and substituting . We obtain



### Design of simulation program – null and power calculations for a fixed sample size

We perform simulations using 100,000 iterations to verify (i) the nominal significance levels under the null hypothesis; and (ii) the asymptotic power calculations provided by equation (2). We use a 2^7 ^factorial design [30] in which we set lower and upper bounds for each set of parameters. In the simulations, we consider both di-allelic and tetra-allelic loci. For each simulation, both the affected and unaffected genotype frequencies are in HWE. For the power simulations using di-allelic loci, the genotype frequencies are specified as follows using a parameter *p*: for the affected group, *p*_01 _= (1 - *p*)^2^, *p*_02 _= 2*p*(1 - *p*), *p*_03 _= *p*^2^, and for the unaffected group, *p*_11 _= (1 - *p *- 0.1)^2^, *p*_12 _= 2(*p *+ 0.1)(1 - *p *- 0.1), *p*_13 _= (*p *+ 0.1)^2^. That is, the SNP minor allele frequency in the unaffected population is equal to the sum of the SNP minor allele frequency in the affected population (*p*) and 0.1. For the null simulations, both the affected and unaffected groups have genotype frequencies as specified above for *p*_0*j*_, *j *∈ {1,2,3}. Our parameter settings for the factorial design are shown in table 1.

For the tetra-allelic loci, the parameter settings are the same as for the di-allelic loci with the exception of the affected and unaffected genotype frequencies. For the tetra-allelic loci, we let *p *= 0.25 and specify the genotype frequencies for power simulations as follows using a parameter *d*. For the affected population, the probability of a homozygous genotype is *p*^2^+*d*(0.03) and the probability of a heterozygous genotype is 2*p*^2 ^- *d*(0.02), where *d *= 1,2. For the control group, the probability of a homozygous genotype is 0.0625 and the probability of a heterozygous genotype is 0.125. For null simulations, we set *d *= 0.

Here, we briefly describe the algorithm used to simulate our phenotype and genotype data for each replicate of a particular simulation. Note that a simulation is completely described by the each of the 7 parameter settings provided in table 1. For each individual in each replicate, we first randomly assign the individual an affection status (affected or unaffected) using the disease prevalence *K*. We then randomly assign the individual a genotype conditional on the affection status using the conditional probabilities *p*_0*j *_and *p*_1*j*_. Once affection status and genotype are determined, we then randomly assign case or control status using the individual's affection status and the phenotype misclassification probabilities. Within each replicate, we repeat this procedure until we have the specified number of cases and controls. Because of the low prevalence, we invariably reach our required number of controls much more quickly than we reach our required number of cases. In such situations, we simply ignore all assigned control individuals after reaching our required number, and keep collecting cases until we achieve that required number.

### Cost functions

We demonstrate how to compute the sample size cost coefficient of phenotype misclassification to gain insight into which type of misclassification requires the greater increase in sample size for fixed power. Let *λ *equal the non-centrality parameter when there is no phenotype misclassification and let λ* equal the non-centrality parameter in the presence of phenotype errors. To find the sample size adjustment needed to maintain constant power, we set *λ *= λ*. We considered this condition previously when studying the cost of genotype error [8]. Let  and . Then the condition *λ *= λ* may be rewritten as  or . Though the cost of misclassification for cases is mathematically defined as the ratio /*N*_*A*_, we instead consider the reciprocal ratio *N*_*A*_/ because the latter allows for more straightforward computation. We approximate *N*_*A*_/ using a first-order Taylor Series expansion centered at (*θ*, *φ*) = (0,0). We obtain . Here, (∂/∂*θ*)[*f*]|_(0,0) _is the partial differential operator (with respect to *θ*) acting on the function *f *and evaluated at the point (0,0). An identical definition holds for (∂/∂*φ*)[*f*]|_(0,0)_.

Since , the previous equation can be rewritten as , where . We note that because , . We let .

### Minimum sample size requirements in presence of phenotype misclassification – Alzheimer's disease ApoE example

We determine the minimum sample size necessary to maintain a constant power of 95% at the 5% significance level using formula (3) and considering estimated genotype frequencies from a recently published genetic association analysis of Alzheimer's Disease (AD) cases and controls genotyped at the ApoE marker locus [9]. In most populations there are three alleles at the ApoE locus. Conventionally, they are denoted *ε*_2_, *ε*_3_, and *ε*_4 _and we label them 2, 3, and 4 respectively in this work. In a well known and often replicated association finding, every copy of the 4 allele in a person's genotype increases that person's risk of getting late-onset AD by a factor of 2.5–3 [31]. Furthermore, recently published estimates of prevalence for Alzheimer's Disease in the US hover around the 2% range [32]. Thus, for our sample size calculations, we assume a prevalence *K *= 0.02.

If we index the six genotypes as 1 = 22, 2 = 23, 3 = 24, 4 = 33, 5 = 34, 6 = 44, then the genotype frequency values we use for our sample size calculations (taken from our previous work [9]) are:

*p*_01 _= 0.019, *p*_11 _= 0.000, *p*_02 _= 0.057, *p*_12 _= 0.118, *p*_03 _= 0.019, *p*_13 _= 0.024, *p*_04 _= 0.465, *p*_14 _= 0.699, *p*_05 _= 0.344, *p*_15 _= 0.159, *p*_06 _= 0.096, *p*_16 _= 0.000.

As it has been documented that phenotype misclassification in Alzheimer's Disease may run as high as 15% or more [19], we consider phenotype misclassification values 0 ≤ *θ*, *φ *≤ 0.15, in increments of 0.01. It is assumed that there are equal numbers of cases and controls (R* = 1).

## Authors' contributions

BJE performed all analyses and wrote the majority of the original manuscript. CH wrote all computer code for simulations. MAL wrote portions of the manuscript and contributed to the development of the results to be presented. SJF and DG formulated the original research question and supervised every stage of the research. They also re-wrote significant portions of the revised manuscripts.

## Appendix

Here, we derive formulas for the case and control genotype frequencies, , , in terms of the affected genotype frequencies *p*_0*j*_, the unaffected genotype frequencies *p*_1*j*_, the disease prevalence *K*, and the misclassification error probabilities, *θ *and *φ*. Zheng and Tian derived similar results in a genetic-model based framework [14].

 = Pr(genotype = *j *| case) = Pr(genotype = *j*, case)/Pr(case)

= [Pr(genotype = *j*, case, affected) + Pr(genotype = *j*, case, unaffected)]/Pr(case)

= [Pr(genotype = *j *| case, affected) Pr(case | affected) Pr(affected) + Pr(genotype = *j *| case, unaffected) Pr(case | unaffected) Pr(unaffected)]/[Pr(case | affected) Pr(affected) + Pr(case | unaffected) Pr(unaffected)]

= [*p*_0*j *_(1 - *θ*)*K *+ *p*_1*j*_*φ*(1 - *K*)]/[(1 - *θ*)*K *+ *φ*(1 - *K*)].

 = Pr(genotype = *j *| control) = Pr(genotype = *j*, control)/Pr(control)

= [Pr(genotype = *j*, control, affected) + Pr(genotype = *j*, control, unaffected)]/Pr(control)

= [Pr(genotype = *j *| control, affected) Pr(control | affected) Pr(affected) + Pr(genotype = *j *| control, unaffected) Pr(control | unaffected) Pr(unaffected)]/[Pr(control | affected) Pr(affected) + Pr(control | unaffected) Pr(unaffected)]

= [*p*_0*j*_*θK *+ *p*_1*j*_(1 - *φ*)(1 - *K*)]/[*θK *+ (1 - *φ*)(1 - *K*)].
